# Inhibition and potential treatment of colorectal cancer by natural compounds *via* various signaling pathways

**DOI:** 10.3389/fonc.2022.956793

**Published:** 2022-09-08

**Authors:** Mingchuan Wang, Xianjun Liu, Tong Chen, Xianbin Cheng, Huijie Xiao, Xianglong Meng, Yang Jiang

**Affiliations:** ^1^ Department of Gastrointestinal Colorectal and Anal Surgery, The China-Japan Union Hospital of Jilin University, Changchun, China; ^2^ College of Food Engineering, Jilin Engineering Normal University, Changchun, China; ^3^ Department of Thyroid Surgery, The Second Hospital of Jilin University, Changchun, China; ^4^ Department of Burns Surgery, The First Hospital of Jilin University, Changchun, China

**Keywords:** colorectal cancer, natural compounds, signaling pathway, flavonoids, polyphenol

## Abstract

Colorectal cancer (CRC) is a common type of malignant digestive tract tumor with a high incidence rate worldwide. Currently, the clinical treatment of CRC predominantly include surgical resection, postoperative chemotherapy, and radiotherapy. However, these treatments contain severe limitations such as drug side effects, the risk of recurrence and drug resistance. Some natural compounds found in plants, fungi, marine animals, and bacteria have been shown to inhibit the occurrence and development of CRC. Although the explicit molecular mechanisms underlying the therapeutic effects of these compounds on CRC are not clear, classical signaling transduction pathways such as NF-kB and Wnt/β-catenin are extensively regulated. In this review, we have summarized the specific mechanisms regulating the inhibition and development of CRC by various types of natural compounds through nine signaling pathways, and explored the potential therapeutic values of these natural compounds in the clinical treatment of CRC.

## Introduction

Natural compounds are chemical substances found in plants, fungi, marine animals, and bacteria that exhibit significant pharmacological effects. Natural compounds can be classified according to their chemical structure as follows: proteins, polypeptides, amino acids, nucleic acids, various enzymes, saccharides, resins, colloids, lignin, vitamins, fats, oils, waxes, alkaloids, volatile oils, flavonoids, glycosides, terpenoids, organic acids, phenols, quinones, lactones, steroids, tannins, antibiotics, and other naturally occurring chemical components. Some natural compounds have the ability to modulate signaling pathways and regulate the expression of genes involved in cell cycle regulation, cell differentiation, and apoptosis (1). One of the most widely researched of these functions is the anti-tumor effect of natural compounds (1).

Colorectal cancer (CRC) is a malignant tumor originating from the colorectal mucosa. It is one of the most common clinical malignant tumors worldwide, with more than 387,600 new cases and 187,100 CRC-related deaths in China, representing the fifth (8.01%) and fourth (9.87%) highest mortality and incidence rates among all cancers in 2015 (2). Thus, reducing the incidence and mortality of CRC is an urgent and important clinical issue. Current CRC treatment methods are based on surgery and chemotherapy, with the most common chemotherapeutic agents being platinum derivatives (oxaliplatin), antimetabolites (capecitabine, 5-fluorouracil (5-FU)), topoisomerase inhibitors (irinotecan), and Tegafur/uracil (UFT) (3). Among these, the first-line chemotherapy drug for CRC treatment is 5-FU (4); however, the overall response rate to 5-FU monotherapy in advanced CRC is limited to 10–15% (5). Moreover, chemotherapy involves different degrees of side effects. The most common adverse effects of 5-FU include vomiting, diarrhea, mucositis of the oral cavity, headaches, skin pruritus, anemia, cardiotoxicity, agranulocytosis, alopecia (hair loss), photosensitivity, hand-foot syndrome, depression, and anxiety (6).

Therefore, the development of anti-tumor drugs that exhibit low toxicity and less potential to develop drug resistance has become the focus of current CRC research, with many researchers turning their attention to natural compounds. Abundant previous research has shown that various types of natural compounds can inhibit CRC. In this review, we discuss the specific mechanisms by which natural compounds inhibit CRC by describing the overall role of several classical signaling pathways in the application of natural compounds to CRC. The anti-cancer activity and clinical potential of natural compounds are reviewed at cellular, animal, and clinical levels.

## Nuclear factor kappa B signaling pathway

### Polyphenolic compounds

Epigallocatechin gallate (EGCG) is a polyhydroxy phenolic compound extracted from tea, which mainly exists in green tea and raw pu-erh tea. Modern medicine has confirmed that EGCG can prevent the occurrence of cardiovascular disease and inhibit the growth and metabolism of tumors (7). EGCG induces p21 expression by enhancing p21 promoter activity (8), releases adenosine by disrupting the folic acid cycle (9), and inhibits CRC cell proliferation (10) and migration (11) by the NF-κB signaling pathway. miRNAs have the ability to regulate biological processes and play a crucial role in the occurrence and development of cancer (12). EGCG can enhance the sensitivity of CRC cells to 5-FU chemotherapy through the NF-κB/miR-155-5p/MDR1 axis (13). According to previous animal models, EGCG inhibits N,N’-dimethylhydrazine-induced colon tumor development through its antioxidant and anti-inflammatory potential (10, 14). In addition, EGCG can be used in combination with sodium butyrate to induce CRC cell cycle arrest and DNA damage (15). Notably, a clinical study involving 32 volunteers reported that daily oral administration of EGCG (800 mg) regulates targeted biomarkers associated with CRC, including NF-κB. (16). These studies reveal the need for further investigation into EGCG as a potential chemopreventive agent for CRC.

As another polyphenol, the main sources of natural resveratrol (RES) are *Polygonum cuspidatum* and *Vitis*; however, RES is also a bioactive ingredient in wine and grape juice, and easily absorbed orally. RES exhibits various pharmacological effects such as anti-tumor, anti-cardiovascular disease, anti-inflammatory, antioxidant, and nervous system protective effects, and has great medicinal value and market prospects (17). According to bioinformatic analysis based on molecular docking and molecular dynamics simulations, the molecular interaction between NF-κB and RES can be applied to develop cancer therapies (18). RES also has inhibitory effects on CRC cell lines by regulating NF-κB, which includes increasing sensitivity to 5-FU chemotherapy (19, 20), inhibiting focal adhesion kinase (FAK) activity and enhancing anti-invasive activity (21), or inducing apoptosis through PD-L1 (22). In addition, there is evidence that RES combined with piceatannol (PIC), a polyphenolic compound also found in grapes, can inhibit iNOS expression, inhibit NF-κB activation (23), and further upregulate PD-L1 (22). Recently, with the popularization of nanotechnology in the pharmaceutical field, silica nanoparticles have been used to encapsulate RES, forming MCM-48-Resveratrol. Compared with pure RES, MCM-48-Resveratrol can more effectively induce CRC apoptosis (24).

Curcumin (CCM) is a diketone hydrophobic polyphenol derived from the dried rhizome of the ginger plant, *Curcuma longa L.* CCM has long played an important role in the treatment of inflammatory-mediated diseases and begun to attract additional attention because of its low toxicity effects in multiple animal and clinical experiments (25, 26). A number of studies have shown that CCM can significantly inhibit CRC through NF-κB during *in vivo* and *in vitro*, for example, by inducing apoptosis and inhibiting metastasis (27). Its therapeutic effect is comparable to that of 5-FU (28), and its combined use with conventional chemotherapeutic drugs such as 5-FU can enhance the effect of chemotherapy (29); thus, it has substantial potential as an anti-chemotherapy-resistant drug for colon cancer treatment. However, high hydrophobicity, poor oral absorption, and fast metabolism limit the clinical use of CCM. To solve these problems, researchers have used nanotechnology to treat CCM, generating a variety of derivatives that retain or even enhance its anti-cancer activity, such as Theracurmin, which boasts improved water solubility (30). These derivatives have greatly expanded the clinical application prospects of CCM (31). Notably, regulation of the NF-κB pathway by CMM was confirmed in a clinical trial in which the combined use of anthocyanins and CCM significantly decreased the expression of NF-κB (32).

### Flavonoids

Kaempferol, extracted from *Prunus mume*, is a flavonoid compound that has attracted widespread attention because of its anti-cancer, anti-inflammatory, antioxidant, anti-bacterial, anti-viral, and other effects. According to both animal and cellular experiments, kaempferol significantly inhibits the growth, migration, and invasion of CRC cells through the RelA/NF-κB signaling pathway, as determined by network pharmacology and molecular docking (33). Moreover, kaempferol can regenerate chemosensitivity in 5-FU-resistant LS174-R cells (34), making it a potentially effective therapy for CRC. Moreover, delphinidin, which is a type of flavonoid abundant in blueberries, inhibits the proliferation of human colon cancer cells (HCT-116) through the NF-κB pathway (35). Furthermore, silibinin can reduce the protein expression levels of various NF-κB regulatory molecules such as Bcl-2, COX-2, iNOS, VEGF, and matrix metalloproteinases (MMPs) (36). Baicalin, a flavonoid compound extracted from the traditional Chinese herbal medicine *Scutellaria baicalensis Georgi*, can exert therapeutic effects by mediating oxidative stress, inflammation-induced downstream apoptosis, and immune response pathways (37).

### Other chemicals

As a quinonemethide triterpenoid extracted from *Celastraceae* and *Hippocrateaceae* species, pristimerin has potent anti-cancer effects (38). Specifically, pristimerin has significant cytotoxic and proliferation inhibitory effects on CRC cell lines. Cell cycle analysis revealed that pristimerin can induce G1 phase arrest, which is closely associated with the reduced expression of cyclin D1 and cyclin-dependent kinases (CDK4 and CDK6) and the induction of p21 (39, 40). Furthermore, the results of Azoxymethane (AOM)/Dextran sulfate sodium (DSS)-induced animal models showed that pristimerin inhibits tumor growth mainly by inhibiting NF-κB activity in tumor tissues (41). Among the triterpenoids, Raddeanin A from the sea anemone *Raddeana Regel* also shows the potential to inhibit the invasion and metastasis of CRC through the NF-κB pathway (42). Wang (43) reported that Raddeanin A inhibits the NF-κB pathway by reducing the phosphorylation of IKBα, thereby inducing the subsequent mitochondrial apoptosis pathway.

Organosulfur compounds in garlic also induce apoptosis in colon cancer cells by modulating NF-κB (44). In SW480 cells and an AOM/DSS-induced mouse CRC model, diallyl disulfide inhibits Glycogen synthase kinase-3β (GSK-3β), a positive regulator of NF-κB, thereby suppressing inflammation and tumors (45). Diallyl sulfide, diallyl disulfide, and diallyl trisulfide also inhibit the migration and invasion of COLO205 cells, with diallyl sulfide exhibiting the greatest effect (46). There have also been reports that allicin increases CRC cell sensitivity to X-ray radiotherapy (47).

### Summary

The relationship between the transcription factor NF-κB pathway, inflammation, and cancer has been extensively evaluated (48–50). In colon cancer, chronic inflammation is an important predisposing factor. Therefore, the NFκB signaling pathway plays an important role in tumor biology, regulating key processes in the occurrence and development of various cancers. These processes include promoting cell proliferation (by regulating cyclin D, c-Myc, and IL-6, which regulate growth-promoting signals), inhibiting apoptosis (by inhibiting apoptotic genes including Bcl-2 and BclxL transcription), promoting angiogenesis (inducing VEGF expression), promoting tumor invasion (through E-selectin and MMPs), promoting generation of the epithelial mesenchymal transition (EMT) and colon cancer stem cells (CSCs), and mediating tumor drug resistance.

**Figure 1:**
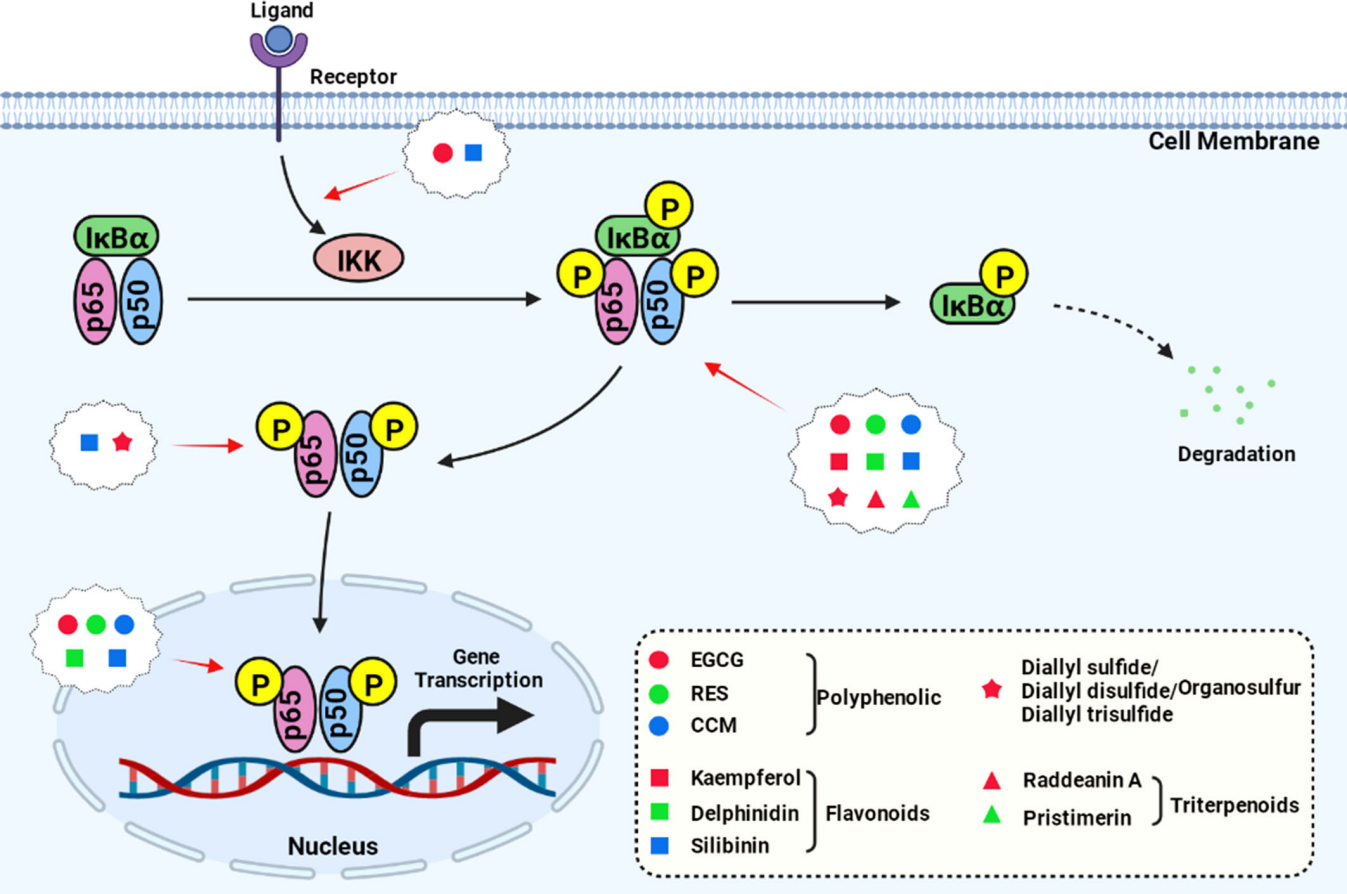
Natural compounds act on the key nodes of the NF-κB signaling pathway.

Some studies have explored the upstream or downstream target of the NFκB pathway and produced more in-depth reports on the anti-cancer mechanism of some natural products (**Figure 1**, created with BioRender.com). For example, RES inhibits the NF-κB pathway and promotes the expression of Sirt1 by inhibiting the activity of FAK (21); EGCG can downregulate glucose-regulated protein 78 (GRP78) expression and activate the NF-κB/mir-155-5p pathway, therefore inhibiting the resistance of colon cancer cells to chemotherapeutic drugs (13); and pristimerin can induce apoptosis and inhibit cell proliferation by inhibiting the Akt/forkhead box protein O (FOXO3a) pathway (41).

However, as these natural products move from the laboratory to the clinic, we also need to consider whether their effective concentrations are acceptable to humans. For example, PIC, although it exhibits G1/S cell cycle arrest and apoptosis-inducing activity in *ex vivo* cultured CRC cell lines at an effective concentration of 50 μM, this concentration is unattainable under physiological conditions (22).

## Wnt/β-catenin signaling pathway

### Flavonoids

Luteolin, a flavonoid component detected in several Chinese herbal medicines, can promote the cycle arrest of HCT-15 cells in the G2/M phase and induce apoptosis (51). Moreover, luteolin was shown to inhibit β-catenin, GSK-3β, and cyclin D1 in a mouse model (52). Apigenin is a flavonoid that is widely distributed in vegetables and fruits grown in the warm tropics, especially celery. Apigenin significantly inhibits the entry of β-catenin into the nucleus, as well as the proliferation, migration, invasion, and organoid growth of CRC cells (53). Apigenin also regulates Akt/mTOR signaling-induced β-catenin degradation during Wnt signaling *via* the autophagy-lysosomal system (54). Genistein shows anti-cancer activity and mainly exists in species of the legume family, such as *Sophora japonica* and *Shandou root*. However, it has exhibited contradictory performance in previous studies on colon cancer and the Wnt/β-catenin signaling pathway. Cellular-level experiments using RKO and DLD1 cell lines revealed that genistein alone cannot inhibit Wnt signaling (55), whereas another report demonstrated that genistein, as an inhibitor of the Wnt/β-catenin signaling pathway, can prevent the development of early colon cancer in animals (56).

Two flavonoid compounds, derricin and derricidin, both extracted from *Lonchocarpus sericeus*, also exhibit anti-colon cancer activity. These compounds can affect the β-catenin distribution and strongly inhibit activity of the canonical Wnt pathway in the CRC cell line (57). Isobavachalcone, a flavonoid compound discovered in *Psoralea corylifolia*, inhibits cell proliferation and induces apoptosis by inhibiting the Akt/GSK-3/β-catenin pathway in CRC (58). Furthermore, taxifolin, a flavonol nonanol widely present in olive oil, grapes, citrus fruits, and onions, can reduce the expression of β-catenin, Akt, and Survivin (59), thereby showing potential as a novel anti-Wnt/β-catenin drug.

### Alkaloids

Piperine, which can be extracted from the dried near-ripe or ripe fruit of *Piper nigrum L.*, has been clinically used as a broad-spectrum anti-convulsant and shows low toxicity and safety in clinical applications (60). Piperine can also inhibit β-catenin nuclear translocation and cell proliferation in the HCT116 CRC cell line (61). Meanwhile, piperine combined with celecoxib combination therapy can synergistically modulate the Wnt/β-catenin pathway, downregulate stemness markers, and enhance the treatment of CT26 homologous Balb/c mice (62). Jatrorrhizine, an alkaloid found in the traditional Chinese herbal medicine *Coptis chinensis*, can inhibit the Wnt signaling pathway by decreasing β-catenin and increasing GSK-3β expression. Wang verified this effect of jatrorrhizine in an HCT-116 nude mouse xenograft model (63). Sanguinarine, an alkaloid of papaveraceae, exhibits negative effects on expression of the Wnt/β-catenin pathway and EMT markers in LoVo cells. The anti-metastatic potential exhibited by sanguinarine may also be attributed to inhibition of the Wnt/β-catenin signaling pathway, suggesting that sanguinarine may be a potential treatment for metastatic CRC (64).

### Terpenoids

Several terpenoids with anti-CRC activity have been discovered in animal models and at the cellular level. RNA-Seq data showed that primimerin can inhibit the Wnt/β-catenin signaling pathway by activating GSK3β (65). Cucurbitacin E, which is widely distributed in dietary plants, can reduce the expression of β-catenin when combined with 5-FU; moreover, transcription factor activating enhancer-binding protein 4 (TFAP4) is the intracellular target of cucurbitacin E (66). Diterpenoid 11α, 12α-epoxyleukamenin E (EPLE), extracted from *Salvia miltiorrhiza*, is a novel Wnt signaling inhibitor and a potential candidate for further preclinical evaluation as a CRC treatment option (67). EPLE mediates the downregulation of Wnt by inhibiting the target genes c-Myc, Axin2, and Survivin, with Wnt signaling identified as the main and specific target of EPLE. Furthermore, the triterpenoid Raddeanin A, may downregulate Wnt/β-catenin signaling *via* inhibition of p-lipoprotein receptor-related protein 6 (LRP6), activation of Akt inactivation, removal of GSK-3β inhibition, and suppression of β-catenin (43).

### Other chemicals

In addition to the aforementioned major classes of compounds, other natural active products show considerable inhibition of Wnt/β-catenin signaling, such as sulforaphane (SFN) and phenethyl isothiocyanate, which are rich in cruciferous vegetables. SFN can inhibit β-catenin degradation and induce to form closed chromatin associated nuclear β-catenin (68). Moreover, phenethyl isothiocyanate can become a potent inhibitor of colorectal cancer stem cells (CSCs) by targeting the Wnt/β-catenin pathway (69). Parthenolide, found in feverfew, inhibits the activity of Wnt signaling by directly acting on ubiquitin-specific peptidase 7 and destabilizing β-catenin, which indicates that parthenolide is a target anti-cancer agent for aberrant ubiquitin-specific peptidase 7/Wnt signaling (70). Physalin F, a steroid in *Physalis*, can inhibit Wnt/β-catenin signaling by modulating the ubiquitination and degradation of β-catenin in a Yes-associated protein (YAP)-dependent manner (71). Another compound in *Physalis*, 4β-Hydroxywithanolide E, can significantly inhibit tumor growth in HCT116 xenografts by suppressing the Wnt/β-catenin signaling; this compound also has potential as a novel Wnt signaling inhibitor (72).

### Summary

The Wnt/β-catenin signaling pathway transmits growth-stimulating signals. Approximately 90% of CRC cases are associated with genetic mutations in the adenomatous polyposis coli (APC) gene or other key components of the Wnt signaling pathway (73), and β-catenin accumulation in the nucleus is associated with poor prognosis in patients with CRC (74).

**Figure 2:**
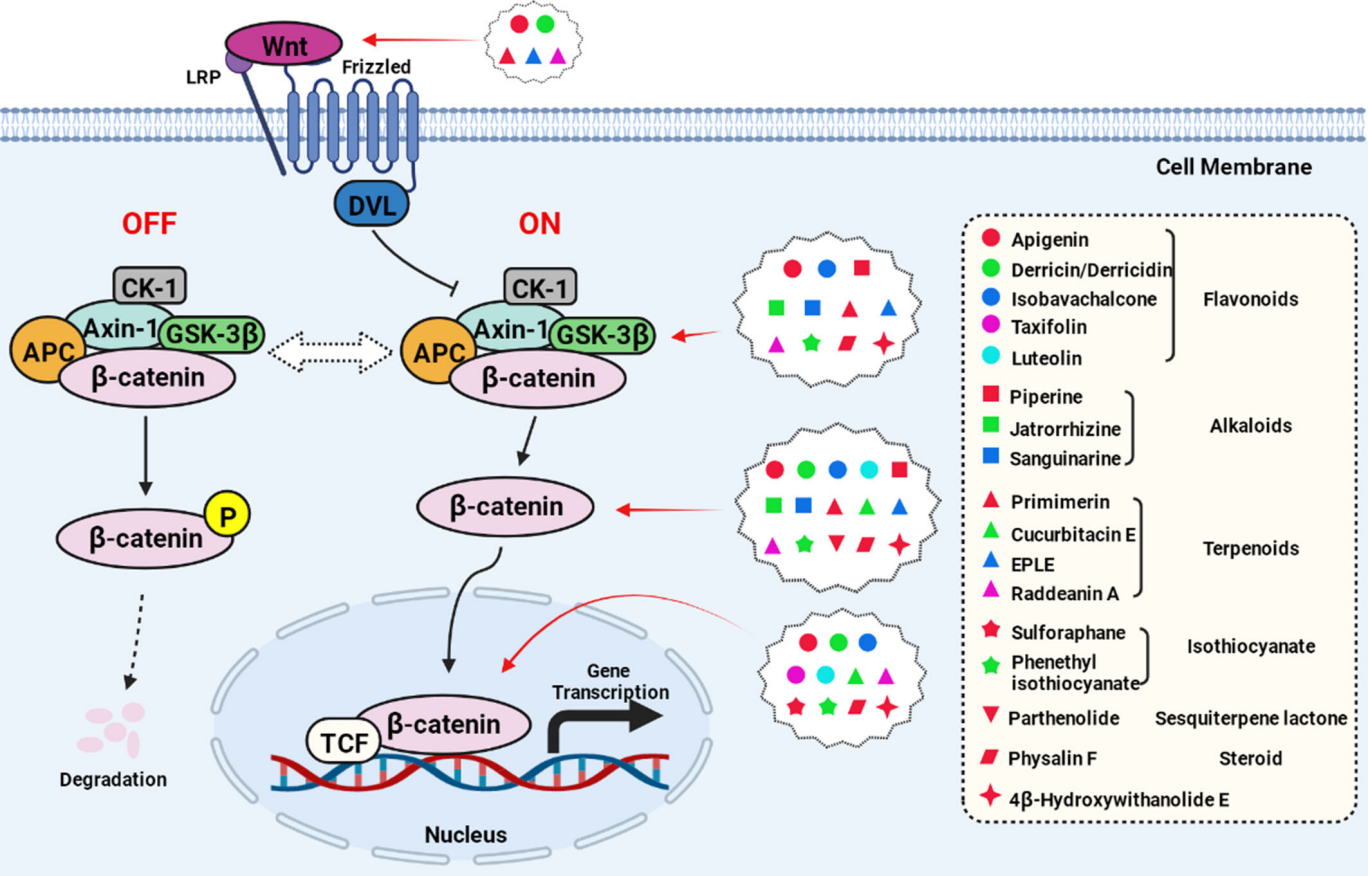
Natural compounds act on the key nodes of the Wnt/β-catenin signaling pathway.

The most common targets of the natural compounds reviewed in this section involve the regulation of cytoplasmic proteins, including β-catenin, GSK-3β, Axin, and APC (**Figure 2**, created with BioRender.com). Almost all the natural compounds mentioned above exhibit inhibitory activity against β-catenin, with some compounds able to regulate the expression of GSK-3β including flavonoids (luteolin, apigenin) and alkaloids (jatrorrhizine, sanguinarine). Compounds that inhibit the expression of Axin include the diterpenoid EPLE and the alkaloid sanguinarine.

In addition to the targets present in this pathway, previous studies have also reported the anti-cancer effects of some natural compounds through upstream or downstream targets. For example, parthenolide can directly interact with ubiquitin-specific peptidase 7 to inhibit the Wnt pathway (70). Moreover, Physalin F is involved in the degradation of β-catenin according to YAP, a transcriptional co-activator in the Hippo pathway (71). Furthermore, cucurbitacin E is a potential inhibitor of the transcription factor TFAP4 that can block the overexpression of β-catenin (66), and Raddeanin A can inhibit β-catenin transcriptional activation through inactivation of the Wnt coreceptor LRP6 (43).

In the process of developing natural products as drugs, the effective concentration is an important consulting index. Among the literature reviewed here, the natural products capable of inhibiting CRC cell proliferation at low effective concentrations include EPLE (2 μM), which dose-dependently inhibits endogenous Wnt signaling and induces apoptosis (67), cucurbitacin E (1 μM), which can increase the apoptosis rate of cancer cells combined with oxaliplatin chemotherapy from 44.36% to 85.3% (66), and Raddeanin A (1.2 μM), which significantly reduces the nuclear level of β-catenin after 6 h of treatment (43). However, these concentrations are at the cellular level. In the future, more specific pharmacological experiments are required to determine the effective concentration of these compounds for CRC inhibition at the animal level.

## Phosphatidylinositol-3 kinase/Akt/mTOR signaling pathway

### Polyphenolic compounds

RES exhibits anti-proliferative and apoptosis-inducing activities on CRC cells by upregulating bone morphogenetic proteins (BMP7) and tensin homolog (PTEN) (75, 76), as well as enhanced sensitivity of CRC cells to cetuximab chemotherapy by upregulating connexin-43 (77). In addition, RES exerts anti-cancer activity through the miR-34a/E2F3/Sirt1 axis (78). A bioinformatics analysis study also found that RES induces the expression of Raf-1 kinase inhibitory protein at the protein level (79).

CCM can regulate the activity of the PI3K/Akt signaling pathway by reducing the phosphorylation of Akt, thereby inhibiting the proliferation of CRC cells and promoting apoptosis (80). The targets and pathways of CCM in the treatment of CRC have been predicted through bioinformatics analysis and molecular docking technology (81). The core targets are AKT1, the epidermal growth factor receptor (EGFR), and STAT3, with AKT1 exhibiting the strongest binding ability to CCM. According to experiments in A/J mice, the combined use of CCM and salsalate more effectively inhibits activation of the PI3K/Akt/mTOR pathway, attenuates the abnormal proliferation of colonic mucosa, and reduces tumor proliferation, whereas CCM or salsalate alone do not inhibit abnormal crypt cell proliferation or tumor multiplicity (82). According to our current knowledge of colonic CSCs, CCM can act as a chemosensitizing drug targeting CSCs, and the combination therapy of chemotherapeutic drugs and CCM may be more effective than single chemotherapeutic drugs. This type of treatment can be achieved by developing an efficient delivery system that can treat CRC *via* nanotechnology-based approaches (83, 84).

EGCG inhibits the activation of the PI3K/Akt signaling pathway in Caco-2 cells by disrupting lipid raft integrity and promoting EGFR degradation (85). Cyclin D1 and p21 have been identified as molecular targets of EGCG in human CRC cells in a dose-dependent manner (8). Moreover, researchers have confirmed the effect of EGCG on PI3K/Akt signaling pathway regulation in dimethylhydrazine-induced rat CRC models, xenografts of nude mice, and mice transplanted with HCT116 cells (10, 86, 87). Compared with EGCG, epicatechin gallate, which is also present in green tea has received relatively little research attention, with only a few studies reporting its ability to induce mitochondrial dysfunction and PI3K/Akt pathway inactivation in CRC cells (88). Both procyanidin B2, a procyanidin in grape seeds (89), and anthocyanins (90) can downregulate the PI3K/Akt pathway and suppress cell proliferation in CRC cells.

### Flavonoids

Several studies have reported various herb-based traditional Chinese medicine formulations (including the JianPi Fu Recipe (91), Fuzheng-Jiedu Decoction (92), Huangqin-Baishao herb pair (93), and Fufang Yiliu Yin (94)), which exhibit the ability to resist colon cancer. Network pharmacology, which predicts active ingredients and potential targets, revealed that the predicted effective ingredients contain a variety of flavonoids, such as kaempferol, isorhamnetin, calycosin, quercetin, wogonin, and nobiletin. Kaempferol can inhibit cell proliferation and induce apoptosis in CRC cells by inhibiting thymidylate synthase or attenuating p-Akt activation (95). Moreover, the combined treatment of Kaempferol and 5-FU can greatly inhibit activation of the PI3K/Akt pathway (34).

Wogonin is the main component of *Scutellaria baicalensis*, and is isolated from the roots and leaves of this plant. It has recently been reported to exhibit a growth inhibitory effect by inhibiting PI3K/Akt in SW48 cells (96). Moreover, wogonin may induce apoptosis in HT-29 colon cancer cells in a p53-dependent manner *via* Akt activation (97). Wogonin also downregulates the expression and glycolysis of HIF-1α by inhibiting the PI3K/Akt signaling pathway, thereby reversing hypoxia-induced drug resistance (98). In a clinical trial, researchers (99) noted that Silybin and regorafenib combination therapy exerts synergistic anti-proliferative and apoptotic effects by blocking the PI3K/Akt/mTOR intracellular pathway, suggesting that such combination therapy may increase the clinical efficacy of regorafenib.

### Other chemicals

Soybean B-group triterpenoid saponins reportedly inhibit Akt activity and induce macroautophagy in human colon cancer cells at physiological concentrations (100, 101). Pristimerin, which is also a triterpenoid, reportedly induces apoptosis and regulates the PI3K/Akt/FOXO3a signaling pathway (39, 41).

According to a previous study, combining salinomycin and SFN in CRC cell lines and xenograft models effectively enhances apoptosis and inhibits the proliferation, migration, and invasion of CRC by inhibiting the PI3K/Akt pathway (102). Therefore, this new combination of salsalate and SFN may also provide a potential strategy for the treatment of CRC. Diallyl sulfide, diallyl disulfide, and diallyl trisulfide in garlic can inhibit COLO205 cell proliferation through multiple pathways, including the PI3K/Akt pathway (46). FraC (RGD-peptide fragacea toxin C) contained in strawberry anemone is significantly toxic to HCT-116 cells harboring the Phosphatidylinositol-4,5-bisphosphate 3-kinase, catalytic subunit alpha (PIK3CA)/Kirsten rat sarcoma viral oncogene homolog (KRAS) mutation, but less toxic to normal cells, which may suggest a new CRC treatment option based on FraC-peptide (103). Recent research on the anti-cancer effects of traditional Chinese herbal medicines has determined that Huangqin-Baishao Herb Pair and Zuojinwan have the potential to inhibit the development of CRC through the PI3K/Akt/mTOR pathway; however, the effective components that may inhibit the development of CRC have not yet been determined (93, 104).

### Summary

Different upstream and downstream genes of the signaling pathway are associated with the development of CRC. In colon cancer, an abnormally activated PI3K/Akt pathway can inhibit p53-independent apoptosis by phosphorylating mTOR and its downstream regulators p70S6K, 4EBP1, etc. (105); this pathway can also involve in regulating VEGR expression in tumor angiogenesis (106) and the expression of cyclinD1 and p21 (107).

**Figure 3:**
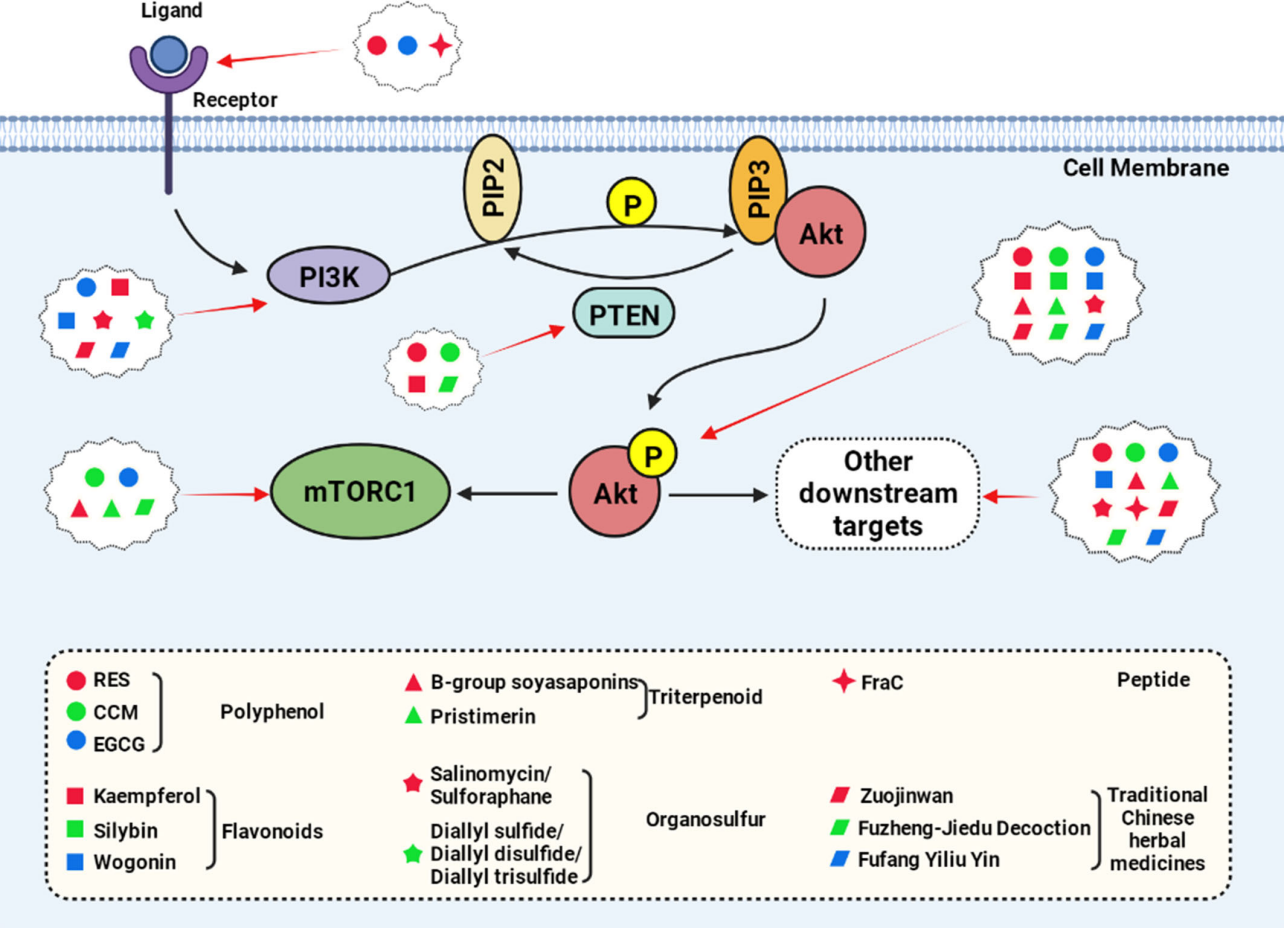
Natural compounds act on the key nodes of the PI3K/Akt signaling pathway.

Multiple natural active products can inhibit Akt phosphorylation, including polyphenols (RES, EGCG, CCM), flavonoids (Kaempferol, wogonin, etc.), and triterpenoid (**Figure 3**, created with BioRender.com).

## Mitogen-activated protein kinase signaling pathway

### Flavonoids

Chemopreventive activity of apigenin in CRC cell lines may be mediated by its ability to modulate the MAPK pathway (108). For example, Zhang observed synergistic inhibition of the proliferation and metastasis of CRC cells by apigenin in combination with chrysin, which is abundant in poplar plants, and inhibited activity of the P38-MAPK/Akt pathway by downregulating p-P38 and p-Akt (109). Kaempferol can also modulate multiple signaling pathways, including MAPK, and can be used alone or in combination with 5-FU to overcome colon cancer drug resistance in cell-level assays and bioinformatics analysis studies (34, 110). Meanwhile, Choi (111) confirmed that kaempferol increases the activation of p-P38 MAPK in HCT116 and HCT15 cells. Anthocyanin, an important class of flavonoids, can potentially mediate CRC cell apoptosis by activating P38-MAPK (112) and inhibiting MMP-2 and MMP-9 activity (90). Silibinin, a flavonoid compound with good anti-cancer ability, can produce anti-cancer effects by regulating the expression of genes related to apoptosis, inflammation, and MAPK pathway proteins, and exhibits higher efficacy when combined with rutin, which is another flavonoid active substance (113). The combination of 5-FU and silibinin can inhibit the dual activation of PI3K/MAPK and CD44v6 to attenuate the activity of colon CSCs, thereby exhibiting potential as a molecular targeted drug (114).

### Polyphenolic compounds

RES can exhibit anti-CRC activity through the MAPK pathway, for example, by activating the P38-MAPK in a bone morphogenetic protein receptor (BMPR)-dependent manner (115), and can decrease CRC cell survival through the P38-MAPK/c-Myc pathway (22). Bioinformatics analysis has revealed that RES can induce the expression of Raf-1 kinase inhibitory protein, thereby inhibiting the MAPK pathway (79). Mohapatra reported the synergistic effect of RES with 5-FU (116), and Wang found that RES can sensitize CRC to cetuximab by upregulating connexin-43 (77).

Studies have also revealed the anti-cancer effect of EGCG in the MAPK pathway. For example, EGCG induces apoptosis and P38-MAPK phosphorylation, thereby downregulating EGFR expression in colon cancer cells (117, 118). Furthermore, Inaba suggested that EGCG-induced P38-MAPK phosphorylation inhibits CRC cell growth by mediating apoptosis rather than regulating the cell cycle (119). Another study showed the involvement of c-Jun N-terminal kinase (JNK) in EGCG-induced apoptosis (120).

CCM inhibits colon cancer cell migration and invasion by inhibiting heparinase expression and P38 phosphorylation, which might be related to the P38-MAPK signaling pathway (121, 122). Moreover, CCM can inhibit insulin-stimulated MAPK and MEK phosphorylation in CRC cells. CCM can also mediate CRC cell apoptosis through JNK signaling, but not through P38 or ERK signaling (123). Furthermore, Sarco/endoplasmic reticulum calcium transport ATPases (SERCA) enzymes play important roles in several signal transduction pathways controlling proliferation, differentiation, and apoptosis (124). Among these enzymes, SERCA2 is involved in the malignant progression of CRC. Furthermore, the CCM analog F36 shows strong anti-cancer activity in CRC cells by targeting SERCA2 (125). Similarly, 6-gingerol, another class of compounds found in Ginger, has potential as a chemotherapeutic drug as it can inhibit the phosphorylation of ERK1/2 and JNK MAP kinases, as well as the activation of AP-1 transcription factors (126).

### Other chemicals

Previous studies on colon cancer cell lines and nude mouse xenograft models have found that α-mangostin has an anti-cancer effect through regulation of the MAPK/ERK1/2 signal pathway (78, 127). Additionally, norcantharidin (NCTD) is a derivative of cantharidin, the main anti-cancer component of traditional Chinese medicine, Cantharides, which is hydrolyzed to remove dimethyl. Studies have found that NCTD has anti-colon cancer activity and can regulate the MAPK signaling pathway (128). Chen used a CRC xenograft model to show that NCTD reduces the expression of p-JNK and p-ERK in the MAPK pathway and exhibits anti-metastatic and anti-angiogenic activities (129). Moreover, Zhang discovered that NCTD inhibits tumor angiogenesis by blocking VEGFR2/MEK/ERK signaling but has little effect on the phosphorylation of P38-MAPK, Akt, or Cox-2 expression (130). This may indicate that NCTD could play a role in anti-cancer therapy as an anti-metastatic and anti-angiogenic agent.

Alantolactone, which is a plant-derived sesquiterpene lactone, exhibits various pharmacological activities, such as anti-bacterial, anti-inflammatory, and anti-viral effects. Alantolactone also shows anti-proliferative and apoptosis-promoting potential by activating the MAPK-JNK/c-Jun signaling pathway in human CRC cells (131). Moreover, the combined treatment of alantolactone and oxaliplatin shows improved anti-tumor activity (132), making it a potential treatment strategy for colon cancer.

Researchers have also discovered active ingredients from a variety of Chinese herbal medicines that can regulate MAPK signaling and inhibit colon cancer. For example, imperatorin can inhibit HIF-1α protein synthesis by downregulating mTOR/p70S6K/4EBP1 and MAPK pathways (133). Honokiol, which is extracted from the bark and leaves of the Chinese magnolia tree, can downregulate the activity of MAPK, PI3K/Akt, and dynamin-related protein 1 signaling and overcome acquired resistance to cetuximab (134). As the main active compound in many medicinal herbs, oleanolic acid significantly inhibits the activation of PI3K/Akt, mTOR/p70S6K and MAPK signaling (135). Taiwanin E, which is abundant in *Taiwania cryptomerioides*, is an estrogen analog that has recently been shown to inhibit CRC cell migration through the P38-MAPK pathway (136). In addition, the polysaccharide of *Ganoderma lucidum* has been confirmed to induce apoptosis in CRC cells by activating the MAPK/ERK pathway (137) and disruption of autophagosome-lysosome fusion (138).

Among several rocaglaol derivatives isolated from *Dysoxylum gotadhora*, 11k significantly inhibit the MAPK pathway in HCT116 cells (139). Furthermore, *Torilis japonica* extract not only modulates the P38-MAPK signaling pathway, but also induces apoptosis and modulates apoptosis-related proteins in the HCT 116 xenograft model (140). Moreover, the Boletus edulis ribonucleic acid fraction in Boletus edulis is able to inhibit DNA synthesis in HT-29 cells and effectively silence signal transduction of the MAPK/ERK pathway (141).

### Summary

The MAPK cascade is a key pathway for human colon cancer cell survival, dissemination, and drug resistance (142). MAPK signaling can be transduced through three main pathways, namely the ERK, JNK and P-38 (143). In colon cancer cells, all three MAPK signaling pathways are involved in inducing apoptosis in different ways. In addition, the MAPK signaling pathway is involved in cell adhesion, angiogenesis, invasion, and metastasis of colon cancer (144).

However, the mechanism for developing anti-cancer active components from natural products based on this pathway remains unclear. Although a considerable number of active components have recently been reportedly related to the MAPK pathway, only a few studies have been dedicated to molecular mechanism. In addition, very few studies have provided new insights through the correlation of natural products with downstream targets, which allow us to gain a deeper understanding of the mechanisms by which natural products exert anti-CRC effects through the MAPK pathway. We have discussed these newly discovered pathway mechanisms above.

## Janus kinase-signal transducer and activator of the transcription signaling pathway

### Polyphenolic compounds

CCM has preventive and therapeutic effects on CRC through multiple targets and multiple pathways, and has also been studied in the JAK/STAT pathway. A recent study found that CCM can inhibit the expression of Nicotinamide N-methyltransferase (NNMT) and p-STAT3 in CRC cells and attenuate NNMT-induced resistance to 5-FU (145). Bioinformatics analysis identified AKT1, EGFR, and STAT3 as the core targets of CCM in the treatment of CRC (81). In addition, feruloylacetone, which is naturally degraded after CCM heating, can also significantly inhibit STAT3 phosphorylation and protein expression levels in CRC cells (146).

The anti-proliferative and anti-migratory effects of EGCG on CRC can be partially achieved by downregulating the expression of STAT3 (147). Moreover, the combination of CCM and EGCG not only attenuates the transition from normal endothelial cells to tumor endothelial cells induced by tumor conditioning medium by inhibiting the JAK/STAT3 signaling pathway, but also significantly reduces tumor growth and angiogenesis in a PDX mouse model of CRC; in this study, the combined anti-angiogenic effect was superior to that of CCM or EGCG alone (148). Among polyphenolic compounds, RES also reduces the level of activated STAT1 in the nucleus (149). However, there are no other studies of the mechanism by which the JAK/STAT pathway might be regulated in colon cancer.

### Other chemicals

Diallyl disulfide induces apoptosis by upregulating the expression of STAT1 in COLO205 cells (150). The molecular mechanism by which allicin promotes apoptosis and inhibits cell proliferation in HCT116 cells has been linked to STAT3 signaling inhibition (151). Among the flavonoids, wogonin (96) and kaempferol (34) both regulate the JAK/STAT3 signaling pathway in CRC cell lines. In addition, a cinnamaldehyde derivative (CB-PIC) was found to suppress P-glycoprotein expression by inhibiting JAK/STAT3 and PI3K/Akt signaling to overcome drug resistance in chemo-resistant cancer cells, thereby becoming an effective chemosensitizer (152). Furthermore, tea polysaccharides can attenuate the progression of colitis-associated cancer by inhibiting the JAK/STAT3 pathway and the expression of downstream genes, such as IL-6 (153). Dihydrotansinone is a diterpenoid anthraquinone compound contained in traditional Chinese herbal medicines that can downregulate the expression of EGFR mRNA and STAT family proteins, leading to anti-proliferative effects (154). The use of cucurbitacin B in CRC cell lines reduces the expression level of pSTAT3 (155). Furthermore, propolis can enhance the apoptosis of butyrate-sensitive CRC cells and resensitize butyrate-resistant CRC cells by inhibiting PI3K/Akt and JAK/STAT pathway (156).

### Summary

In colon cancer, aberrant IL-6/STAT/SOCS3 signaling may be critical for CRC development and progression (157). A dysfunctional JAK2/STAT3 signaling pathway can induce cancer and inhibit apoptosis (158), promote cancer cell migration by regulating E-cadherin and vimentin expression (159), and promote EMT (160). STAT3 plays a particularly crucial role in the regulation of inflammation (161). Most of the natural products discussed in this review also play a role through STAT3, such as allicin, CCM, EGCG, feruloylacetone, wogonin, kaempferol, dihydrotanshinone, cucurbitacin B, CB-PIC, tea polysaccharides, and propolis. Some natural products such as RES and diallyl disulfide can also resist cancer by interacting with the STAT1 protein.

## TGF-β/SMAD signaling pathway

### Polyphenolic compounds

CCM also exhibits anti-cancer properties *via* regulation of the TGF-β/SMADs signaling pathway. Moreover, CCM can reverse oxaliplatin resistance in CRC by inhibiting TGF-β/SMADs signaling at cellular and animal levels (162). CCM exhibits therapeutic potential to inhibit CRC cell metastasis *via* the interaction of stromal cells and tumor cells (163). Furthermore, CCM can upregulate the expression of TGF-β1 and HES-1 (164). Moreover, RES can inhibit EMT by mediating Snail/E-cadherin expression (165), as well as oncogenic processes and tumor growth, by regulating TGF expression (166). Oxyresveratrol, a natural derivative of RES, can also inhibit human colon cancer cell migration by regulating EMT and miRNA (167). In addition, EGCG can lead to a transient increase in TGF-β2 expression in CRC cells (168).

### Other chemicals

Several other classes of compounds have been reported in recent years. For example, ursolic acid can inhibit CRC cell invasion by modulating the TGF-β1/zinc finger E-box-binding homeobox/miR-200c signaling network (169). SFN can reduce the protein expression and enzymatic activity of ornithine decarboxylase by inducing TGF-β/SMAD signaling (170). Baicalin inhibits endogenous and exogenous TGFβ1-induced EMT in CRC cells by inhibiting the TGFβ/SMAD pathway (171). Oxymatrine, an alkaloid extracted from the Chinese herbal medicine *Sophora flavescens Ait*, has been shown to inhibit the growth of CRC, and regulate the expression of TGF-β1, Smad4, and pSmad2 (172). Finally, tetrandrine is a bisbenzylisoquinoline alkaloid, whose anti-cancer activity has been demonstrated to inhibit PI3K/Akt signaling by upregulating TGF-β1 and reduce PTEN phosphorylation (173).

### Summary

In colon cancer, the TGF-β/SMAD pathway has dual roles. In the early inflammatory response associated with CRC, TGF-β has an inhibitory effect on cancer development. Then, once CRC is identified, the TGF-β pathway promotes the development of cancer (162). An abnormal TGF-β/SMAD pathway can promote the migration of cancer cells by evading immune surveillance, inhibit apoptosis, promote tumor angiogenesis by regulating the expression of VEGF, and induce EMT in late tumor stages (165). Moreover, EMT regulated by the TGF-β/SMAD signaling pathway has been gradually recognized as an important mechanism of tumor chemoresistance. The anti-cancer activity of natural products through the TGF-β/SMAD pathway is most strongly manifested in the inhibition of EMT. Approximately half of the natural products have reported effects, including CCM, RES, oxyresveratrol, baicalin, and oxymatrine.

## Notch signaling pathway

### Natural compounds

CCM combined with cisplatin can promote the apoptosis of HT29 cells by regulating the expression of related apoptosis genes and inhibiting activation of the Notch1 signaling pathway (174). Quercetin and ionizing radiation combination therapy exhibits anti-cancer effects by targeting CSCs and inhibiting Notch1 signaling (175). Cucurbitacin B or cucurbitacin I can inhibit the growth of colon cancer by inhibiting the Notch signaling pathway (176). (-)-gossypol (177) and gossypolone (178), both downregulate Notch/Wnt signaling and inhibit the growth of CRC cells by inhibiting the RNA-binding protein Musashi-1. Moreover, genistein can inhibit the proliferation of several cancer cells and induce apoptosis. In CRC, genistein can inhibit colon cancer cell migration by reversing EMT through inhibition of the Notch1/NF-κB/slug/E-cadherin pathway (179), suggesting its potential as an antimetabolite for colon cancer.

### Other chemicals

Secondary metabolites of lichens are promising biological resources for candidate anti-cancer drugs. For example, physciosporin, a compound isolated from *Pseudocyphellaria granulata*, inhibits colon cancer cells through the Notch signaling pathway (180). Moreover, withaferin-A, a bioactive compound derived from *Withania somnifera*, inhibits Notch-mediated prosurvival signaling, which promotes c-Jun-NH(2)-kinase-mediated apoptosis (181).

### Summary

In colon cancer, Notch signaling is dysregulated throughout CRC tumorigenesis, with both Notch receptors and Notch ligands found to be dysregulated (182–184). Almost 80% of CRC cases have reported high expression of Notch1, Notch2, HES1, Delta1, and Jagged1 proteins (185). Moreover, abnormally high expression of the Jagged1 protein can promote cell proliferation and migration by promoting the expression of Cyclin D1, Cyclin E, and c-myc (186), as well as inducing EMT (187). Similarly, aberrant expression of Notch2 can promote tumor metastasis and EMT. miR-139-5p and its target gene Notch1 modulate CRC sensitivity to 5-FU by regulating the expression of its downstream MRP-1 and Bcl-2 (188), and Notch1 can also promote cancer development by inhibiting the expression of PTEN (189).

## Hedgehog signaling pathway

The HH pathway plays a crucial role in embryonic development, tissue homeostasis, and carcinogenesis (190, 191). Recent studies have found that it also contributes to the resistance of cancer cells to various chemotherapeutic drugs (192). Considering current CRC treatment options, it is crucial to identify new drugs and strategies to help overcome HH-mediated drug resistance.

To date, only a few compounds are reported to inhibit cancer through the HH pathway. Inoscavin A, a pyrone compound isolated from *Sanghuangporus vaninii* extract, inhibits the activity of the HH pathway by inhibiting Smo, thereby inhibiting cell proliferation, promoting apoptosis, and exerting an anti-cancer effect (193). Physciosporin also inhibits colon cancer cells through the HH signaling pathway (180). Garcinone C, a natural xanthone derivative found in the *Garcinia mangostana*, can prevent the growth of colon tumors in both cells and animals through non-canonical HH signaling dependent on Gli1 (194). This suggests that garcinone C may be a good chemopreventive agent against the growth of colon tumors.

## Hippo signaling pathway

As the Hippo pathway plays a critical role in cell proliferation, differentiation, apoptosis, and tumorigenesis, it has become a research hotspot in recent years. Components of the Hippo pathway are deregulated in various tumors, and the expression levels of its major signal transmitters are considered to be one of the prognostic factors in CRC (195). Moreover, EGCG inhibits colon cancer tumor growth by downregulating the sonic hedgehog protein (86).

A few natural compounds have been linked to this pathway in recent years. Chai revealed a novel therapeutic mechanism of cucurbitacin B, namely, inhibition of the proliferation and invasion of CRC cells and promotion of apoptosis *via* inhibition of Hippo-YAP signaling and its downstream target genes Cyr 61 and c-Myc in CRC cells (196). Xue found that fucoidan can activate the Hippo pathway and downregulate the β-catenin pathway to induce tumor cell apoptosis and inhibit tumor growth, and that fucoidan can prevent CRC by regulating intestinal microbiology (197). Additionally, lappaol F, a natural lignan from *Arctium lappa Linné*, inhibits tumor cell growth by inducing cell cycle arrest. Lappaol F was recently recognized as an inhibitor of YAP to exert anti-CRC activity (198). Ginsenoside compound K, a component of ginseng, exhibits an anti-CRC effect by inhibiting the expression of adipocyte-specific phospholipase A2, which could promote the progression of CRC by inhibiting the Hippo signaling pathway (199).

## Concluding remarks

Among polyphenols, CCM, EGCG, and RES exhibit proven anti-cancer effects through various signaling pathways (**Table 1**). Through the regulation of various pathways by EGCG, CCM, and RES, we can gain a deeper understanding of the crosstalk between these pathways and the overall mechanism by which natural compounds exert anti-cancer effects. These three compounds are still widely studied by researchers, especially in relation to NF-κB and PI3K/Akt pathways. With advances in bioinformation technology, many studies have verified the correlation between these three compounds and the pathways discussed in this review, for example, through bioinformatics analysis and molecular docking, with all reporting positive correlations. In addition, researchers have focused on the analogs or metabolites of these three substances, and attempted to discover the smallest amounts of these natural products that can exert anti-cancer effects, which can contribute to the future development of novel anti-cancer drugs based on natural active products. Another large class of natural active products is flavonoids. At present, new flavonoids are extracted from plants every year and subsequently investigated *via* research into anti-cancer treatments. Thus, the articles discussed in this review report a relatively large number of flavonoids. In addition to the safety of natural compounds in the human body, clinical trials have also proven the ability of such compounds to inhibit cancer.

**Table 1:** Summary of main natural compounds involved in different pathways with therapeutic approach to colorectal cancer.

Scientific name	Category	Pathway	Cell lines/model	Target	REF
Curcumin	Polyphenol	JAK/STAT	SW480, HT29, BALB/c nude mice (SW480)	NNMT, p-STAT3	(145)
		MAPK	C57BL/6 mice	SERCA2	(125)
			CT26	HPSE, p-P38, p-STAT5,	(121)
			HCT116	p-JNK, p-p38, p-ERK	(123)
			MC38 (rat source)	p-MAPK, p-MEK,	(122)
			SW480, SW620, Caco2, HCT116	SERCA2, MAPK, AKT, cyclin D1, CDX2	(125)
		NF-κB	HCT-116	NF-κB, MMP-9, E-cadherin, Claudin-3, Fas, FADD, caspase-3, caspase-8	(28)
			HCT-116	IκBα, caspase-8/9/3, PARP, Bax, Bcl-xL, cyclin D1	(29)
			Kunming mice (HCT116)	——	(28)
		Notch	HT29	——	(174)
		PI3K/Akt	A/J Mice	——	(82)
			LoVo	Akt, Bax, Bcl-2, caspase-3	(80)
		TGF-β/SMAD	mice	TGF-β1, HES-1	(164)
			HCT116	——	(162)
			HCT116, MRC-5	ICAM-1, TGF-β3, p-Smad2, cyclin D1, Ki-67, vimentin	(163)
EGCG	Polyphenol	Hedgehog	SW480	PI3K, p-AKT, Smo, Gli-1, MMPs, Bax, bcl-2	(86)
			BALB/c nude mice (SW480)	Bax, E-cadherin, bcl-2, N-cadherin	(86)
		JAK/STAT	SW480, SW620, LS411N	STAT3, caspase-3, PARP, p-STAT3, Bcl-2, MCL-1, Vimentin	(147)
		MAPK	HCT116	p-MAPK, p-ERK, p-JNK, p-p38	(119)
			HT-29	JNK, Caspase-3/9	(120)
			HT-29, HCT-116	p-ERK1/2, p-JNK1/2, p-p38α, p-p38γ, p-p38δ, p-Akt	(117)
			SW480	EGFR, p-p38 MAPK	(118)
		NF-κB	rats	——	(10)
			Wistar rats	NF-κB, COX-2, IL-6	(14)
			Caco-2	TNF-α, IκBα, Akt,	(9)
			HCT-116, DLD-1	GRP78, NF-κB, MDR1, caspase-3, PARP, Bcl-2	(13)
			HCT-116, HT-29, Caco-2	IKK, ERK, PI3K, cyclinD1, p21	(8)
			RKO, HCT-116, HT-29	p65, HDAC1, DNMT1, survivin	(15)
			SW620	p65/RelA, ERK1/2, Caspase-7, TCF, MMP-9	(11)
		PI3K/Akt	rats	——	(10)
			Caco-2	EGFR, MMP-2/9	(85)
			HCT116, HT-29	p-Akt	(87)
			HCT-116, HT-29, Caco-2	PI3K, IKK, ERK, cyclinD1, p21	(8)
			SW480	PI3K, p-AKT, Smo, Gli-1, MMPs, Bax, Bcl-2	(86)
			BALB/c nude mice (SW480)	Bax, E-cadherin, Bcl-2, N-cadherin	(86)
			mice (HCT116)	BNIP3, caspase-3, Bcl-2	(87)
		TGF-β/SMAD	SW837	TGF-β2, IGF, IGF-1R, IGFBP-3, MMP-7, MMP-9	(168)
Resveratrol	Polyphenol	JAK/STAT	HT-29	STAT1	(149)
		MAPK	mice	NF-κB, ERK, STAT3, iNOS	(22)
			HCT-116	p-JNK, p-p38	(116)
			LoVo	BMP9, p38 MAPK	(115)
			SW620	HDAC3/p300, p65, p38MAPK, PD-L1, γH2AX, caspase-3	(22)
		NF-κB	HCT116	TNF-β, IκBα, p65, MMP-9, cyclin D1, Ki-67, CXCR4, caspase-3	(19)
			HCT116	TNF-β, NF-κB, MMP-9, cyclin D1, Ki-67, CXCR4, caspase-3	(20)
			HCT116, SW480	FAK, p50, p65, Sirt1, MMP-9, MMP-13, CXCR4, caspase-3	(21)
			SW620	——	(22)
		PI3K/Akt	DLD-1	E2F3, p-Akt, Sirt1	(78)
			HCT116	Akt1/2, PTEN	(75)
			HCT116	BMP7, Akt1//2/3, PTEN	(76)
			HCT116, CT26	Connexin 43, p-Akt, p-mTOR, IKKα, IκBα, p65,	(77)
			athymic nude mice (HCT116)	——	(75)
			Balb/c mice (CT26), nu/nu nude mice (HCT116)	——	(77)
		TGF-β/SMAD	Min mice	——	(166)
			LoVo	——	(165)
Anthocyanin	Flavonoid	MAPK	HCT-116	IAP	(112)
			HCT-116	MMP-2, MMP-9	(90)
		PI3K/Akt	HCT-116	Akt, MMP-2, MMP-9	(90)
Apigenin	Flavonoid	MAPK	HCT116	p-ERK, p-p38	(108)
			SW480, HCT-116	p-P38, p-AKT, MMP2, MMP9, Snail, Twist, Bax, Bcl-2	(109)
			HCT-116, SW480	LRP5, β-catenin, Axin2, c-Myc, cyclin D1	(54)
			SW480, HCT15	β-catenin, TCF/LEF	(53)
Kaempferol	Flavonoid	JAK/STAT	LS174-R	NF-κB, Akt, STAT3, caspase 3/9, PARP, p21, p27, p53, CDC2	(34)
		MAPK	HCT116, HCT15, SW480	p53, p-p38, MAPK, PARP, caspase-8/9/3, p21	(111)
			LS174-R	NF-κB, Akt, STAT3, caspase 3/9, PARP, p21, p27, p53, CDC2	(34)
		NF-κB	C57BL/6 male mice	——	(33)
			HCT116, Lovo	RelA, Bax, caspase 3/9, EGFR	(33)
			LS174-R	NF-κB, Akt, STAT3, caspase 3/9, PARP, p21, p27, p53, CDC2	(34)
		PI3K/Akt	HCT-8, HCT-116	Thymidine synthase, p-AKT, Bax, Bcl-2	(95)
			LS174-R	AKT, ERK1/2, p38 MAPK	(34)
Silibinin	Flavonoid	MAPK	HCT116	——	(114)
			HT-29	——	(113)
		NF-κB	SW480, LoVo, HT29	p65, p50, IκBα, Bcl-2, COX-2, iNOS, VEGF, MMPs	(36)
			nude mice(SW480,LoVo)	NF-κB, Bcl-2, COX-2, iNOS, VEGF, MMPs	(36)
		PI3K/Akt	SW48, HCT15, SW480	pAKT	(99)
Wogonin	Flavonoid	JAK/STAT	SW1417, SW48, DLD-1, HCT-15, LS-180, CCD-18Co	——	(96)
		PI3K/Akt	HCT116	p-Akt, PI3K, HIF-1α, Glycoly-associated proteins(HKII,PDHK1,LDHA)	(98)
			HT-29	Akt, Bcl-2, Bax	(97)
			SW48	p-PI3K, p-AKT, LC3II, Beclin 1, caspase 3/8/9, Bax	(96)
			mice(HT-29)	——	(97)
Genistein	Flavonoid	Notch	HT-29	Notch1, NF-κB, slug, E-cadherins	(179)
		Wnt/β-catenin	Sprague-Dawley rats	β-catenin, WNT5a, Sfrp1, Sfrp2, Sfrp5, Cyclin D1, c-Myc	(56)
Cucurbitacin B	Triterpenoid	JAK/STAT	HT-29, HCT-116	——	(155)
		Notch	HCT116, SW480, DLD1	——	(176)
			athymic nude mice(HCT116)	——	(176)
		Hippo	SW620, HT29	——	(196)
Pristimerin	Triterpenoid	NF-κB	mice	NF-κB	(41)
			HCT-116	TNF-α, IKK, IкB-α	(40)
			HCT-116, COLO-205, SW-620	EGFR, HER2, Erk1/2, Akt, mTOR, NF-κB, caspase-3/8, PARP-1, Bcl-2	(39)
			——	NF-κB	(40)
		PI3K/Akt	BALB/c mice	IκB-α, p-AKT, p-FoxO3a, TNF-α, IL-6, iNOS, COX-2, p2, p21, Bcl-2, Bcl-XL	(41)
			HCT-116, COLO-205, SW-620	p-EGFR, p-HER2, p-Erk1/2, p-Akt, p-mTOR, p-NF-κB, CDK4, CDK6, p21, caspase3/8, PARP-1, Bcl-2	(39)
		Wnt/β-catenin	C57BL/6 mice	Axin2, LEF1, LRP5, c-Myc, Cyclin D1	(65)
			HCT116, HT-29	Akt, GSK3β, β-catenin, c-Myc, cyclin D1, cox-2	(65)
			Balb/c-nu mice(HCT116)	GSK3β, β-catenin, c-Myc, cyclin D1	(65)
Raddeanin A	Triterpenoid	NF-κB	SW480, Caco-2, HT-29, LOVO	p-LRP6, IкB-α, GSK-3β, β-catenin	(43)
		Wnt/β-catenin	SW480, LOVO	p-LRP6, AKT, GSK-3β, β-catenin, IKBα, c-Myc, CyclinD1	(43)
			nude mice (SW480)	p-LRP6, p-AKT, GSK-3β, β-catenin, IKBα	(43)
Ursolic acid	Triterpenoid	MAPK	HCT15, CO115	p-Akt	(200)
			HT-29	EGFR, p-ERK1/2, p-p38, MAPK, p-JNK, Bcl-2, Bcl-xL, caspase 3/9	(201)
			SW480, SW620, LoVo, RKO	MEK1/2, ERK1/2, p-38, JNK, AKT, IKKα, IκBα, Bcl-xL, Bcl-2, survivin, caspase-3/8/9, KRAS, BRAF	(202)
			mice(SW620)	——	(202)
		TGF-β/SMAD	HCT116, HCT-8	TGF-β1, p-Smad2/3, p-focal, Adhesion kinase, ZEB1	(169)
Sulforaphane	Isothiocyanate	PI3K/Akt	Caco-2, CX-1	PI3K, p-Akt, Bax, Bcl-2, p53, PARP	(102)
			BALB/c nude mice (Caco-2)	——	(102)
		TGF-β/SMAD	Caco-2	——	(170)
		Wnt/β-catenin	SW480, DLD1, HCT116	AXIN2, LGR5	(68)
Allicin	Organosulfur compound	JAK/STAT	mice	——	(151)
			HCT116	STAT3	(151)
		NF-κB	BALB/c mice (CT26)	NF-κB, IKKβ, IκBα	(47)
DADS	Organosulfur compound	JAK/STAT	colo 205	STAT1	(150)
		NF-κB	mice	GSK-3β, NF-κB	(45)
			colo 205	NF-κB, PI3K, Ras, MEKK3, MKK7, ERK1/2, JNK1/2, p38, MMP-2/7/9, COX-2	(46)
			SW480	GSK-3β, NF-κB	(45)
		PI3K/Akt	colo 205	NF-κB, PI3K, Ras, MEKK3, MKK7, ERK1/2, JNK1/2, p38, MMP-2/7/9, COX-2	(46)
Physciosporin	Anotherlichen acid	Hedgehog	DLD1, Caco2, HT29	——	(180)
		Notch	CSC221, DLD1, HT29, HEK293T	——	(180)

Among the many natural compounds, clinical studies on CCM are particularly abundant. For example, a phase II trial study reported that oral CCM is a safe adjunctive drug with added benefits in FOLFOX chemotherapy for patients with metastatic CRC (203). Moreover, treating CCM with liposomes can improve its high hydrophobicity. Another phase I clinical trials have also shown the safety of intravenous liposomal CCM (204). A study using micronized RES, which is called SRT501, found that it was well tolerated in patients with liver metastases from CRC, and had certain clinical benefits (205). A group of clinical trials recruiting patients with colon cancer resection and polypectomy showed that continuous long-term treatment with a combination of daily standard doses of apigenin (20 mg) and EGCG (20 mg) can reduce the recurrence rate of tumors (206). In clinical trials on the efficacy of EGCG, researchers have mostly employed oral green tea preparations. Indeed, oral green tea extract (EGCG, 800 mg/day) or green tea polyphenol preparation Poly E (EGCG, 780 mg/day) is well tolerated by people at high risk of CRC (207). Oral green tea extract can also reduce DNA methyltransferase (DNMT1) and NF-κB mRNA in CRC tissues (16), whereas oral Poly E administration for six months failed to reduce rectal abnormal crypt foci numbers in afflicted subjects (207). However, as this evidence is insufficient to describe the role of EGCG in preventing CRC, longer-term clinical trials are required. The combined use of natural compounds with traditional chemotherapy drugs is worthy of further research attention. Natural compounds have an ability to reduce the side effects and improve the efficacy of conventional chemotherapy, which places a considerable burden on the patient’s body (**Table 2**).

**Table 2:** Clinical trials studied in natural compound for chemoprevention and treatment of colorectal cancer.

Agent	Combination strategy	Volunteer(n)	Phage	Trial No.	Status	Administration	Doses	Main outcomes	REF
Curcumin	FOLFOX	Metastatic colorectal cancer, 12	I	NCT01490996	Completed	Orally	0.5, 1, 2g/day	Safety and tolerability for curcumin in combination with FOLFOX chemotherapy	(208)
Curcumin		Healthy volunteer, 24 (34)	I	FWA 00004969	Completed	Orally	500,1000,2000,4000,6000,8000,10000,12000mg(in single oral dose)	The tolerance in single oral doses up to 12,000 mg appears to be excellent	(209)
Curcumin	FOLFOX	Metastatic colorectal cancer, 27 (28)	II	NCT01490996	Completed	Orally	2g	Safe and tolerable adjunct to FOLFOX chemotherapy	(203)
Curcumin		Familial adenomatous polyposis, 44	II	NCT00641147	Completed	Orally	3000 mg/d (twice per day for 12 months.)	No difference in the mean number or size of lower intestinal tract adenomas between patients given curcumin 3,000 mg/day and those given placebo for 12 weeks.	(210)
Curcuminoid	Anthocyanin	patients with adenomatous polyps of the colon, 29 (35)	II	NCT01948661	Completed	Orally	200mg/d (twice per day for 4–6 weeks)	Combining anthocyanins and curcumin lead to a potentially favorable modulation of tissue biomarkers of inflammation and proliferation in colon adenomas.	(32)
Curcuminoid		CRC stage 3, 67 (72)	II	?	Completed	Orally	500mg/d (twice per day for 8 weeks)	Improve ESR and serum levels of CRP in stage-3 CRC subjects and improve the global quality of life and functional scales compared to placebo.	(211)
Liposomal curcumin		Healthy volunteer,50	I	NCT01403545	Completed	Vessel injection	120 mg/m2 (10 - 400 mg/m2; n = 2 - 6 per group)	Safe up to a dose of 120 mg/m2.	(212)
Liposomal curcumin		Locally advanced or metastatic cancer,32	I	NCT02138955	Completed	Vessel injection	300 mg/m2 over 6 h was the maximum tolerated dose	300 mg/m2 liposomal curcumin over 6 h was the maximum tolerated dose in heavily pretreated patients, with significant tumor marker responses and transient clinical benefit	(204)
GTE (green tea extract)		Risk for CRC, 30 (61)	II	ACTRN12613000097741	Completed	Orally	GTE capsules (800 mg EGCG/d) for 6 weeks	GTE regulates targeted biomarkers related to CRC oncogenesis, specifically genes associated inflammation (NF-κB) and methylation (DNMT1).	(16)
Poly E		Colorectal adenomas or cancer, 32 (39)	II	--	Completed	Orally	1200mg Poly E/d for 6 months(containing 65% EGCG)	Well tolerated, but did not significantly reduce the number of rectal aberrant	(207)
Resveratrol		Resectable colorectal cancer, 20	II	--	Completed	Orally	0.5g RES/d or 1.0g RES/d for 8 days	Resveratrol exerted a small reduction in cell proliferation in colorectal tissue after	(213)
								ingestion,but the biological importance of such a slight decrease is debatable.	
SRT501		Stage IV colorectal cancer, 9	--	NCT00920803	Completed	Orally	5.0 g/d for 10-21 day	i. its daily consumption for 14 days seems to be well tolerated in colorectal cancer patients, ii. Cmax for SRT501 was higher than reported for equivalent dose of non-micronised resveratrol, iii. its ingestion furnished measurable resveratrol levels in a tissue distant to the GI tract (in particular the liver), and these concentrations were accompanied by a significant pharmacological effect.	(205)
EGCG+ apigenin		Patients with resected colon cancer, 87 (160)	--	--	Completed	Orally	20 mg apigenin and 20 mg EGCG for 3-4 years	Reduce the recurrence rate of colon neoplasia in patients with resected colon cancer.	(206)
Curcumin+ quercetin		Familia ladenomatous polyposis, 5	--	--	Completed	Orally	curcumin 1440mg/d+ quercetin 60mg/d for 6 months	Reduce the number and size of ileal and rectal adenomas in patients with FAP without appreciable toxicity.	(214)
Genistein	FOLFOX or FOLFOX-Bevacizumab	Metastatic colorectal cancer, 13 (14)	I/II	NCT01985763	Completed	Orally	60mg/d	Safe and tolerable.	(215)
Isoflavones		Adenomatous polyps, 125(150)	--	--	Completed	Orally	58 g protein powder/d containing 83 mg isoflavones or ethanol-extracted soy-protein powder containing 3 mg isoflavones for 12 months	Supplementation with soy protein containing isoflavones does not reduce colorectal epithelial cell proliferation in the cecum, sigmoid colon, and rectum and increases cell proliferation measures in the sigmoid colon.	(216)
GCP (genistein combined polysaccharide)		Healthy volunteer,8	--	--	Completed	Orally	--	serum concentrations of genistein in the subjects treated with GCP (n = 4) at 3 h after administration were significantly higher than those in the subjects treated with SBE (n = 4).	(217)

It is worth mentioning that immunotherapy by inhibiting immune checkpoints as an anti-cancer method has attracted much attention in recent years. It is regarded as an effective treatment to improve the survival rate of patients with advanced or/and metastatic cancer (218). However, the serious adverse reactions that may be caused by immunotherapy, and the cancer cells themselves may not respond to immunotherapy, seriously restrict the clinical application of immunotherapy. At present, natural compounds have been shown to modulate intracellular biochemical reactions including the regulation of immune checkpoints such as PD-L1 and cytotoxic T-lymphocyte antigen 4 (CTLA-4), through a variety of signaling pathways. These effects have been confirmed in multiple cancer types including colon cancer (219). Previous studies have revealed that NF-κB, PI3K and MAPK signaling pathways were involved in the regulation of CRC *via* PD-L1 by resveratrol, piceatannol and panaxadiol (22, 220). Therefore, it might be predicted that natural compounds combined with immunotherapy will bring us new ideas for cancer therapy. Future clinical trials may validate the synergy of the natural compounds and immune checkpoint inhibitors.

Natural compounds and their potential for clinical applications undoubtedly have many advantages. Nevertheless, many limitations hinder the industrialization of many types of natural compounds:

1. The excellent safety and tolerable safe dose to the human body are certainly advantages of natural compounds; however, in most natural compounds, their safety is ascribed to their easy degradation by the human digestive system. As such, it is difficult to achieve a sufficient blood drug concentration *via* oral administration. Moreover, their decomposition efficiency in the digestive system is difficult to calculate, which further increases the difficulty of selecting an appropriate dose for clinical applications.
2. Most of the natural compounds discussed in this review are derived from food. However, according to epidemiological statistical reports, patients with CRC that ingest these natural compounds through diet alone do not report anti-CRC effects, particularly after surgery or during chemotherapy. Therefore, a method of extracting these natural compounds from animals and plants must be found, and the cost of such extraction must also be considered.
3. The anti-cancer mechanism of many natural compounds has not been well studied, the downstream genes remain unclear, and *in vivo* experiments are lacking. Currently, only research into RES, EGCG, CCM, genistein, and quercetin has achieved clinical levels.
4. The combined use of natural products and existing chemotherapeutic drugs has been the focus of many studies; however, nanotechnology advances in recent years have opened up new prospects for the application of natural compounds. Specifically, combining natural compounds with nanomaterials could overcome several of the shortcomings of natural products, such as high hydrophobicity and rapid degradation, and enable the development of new application forms based on natural compounds, such as intravenous injection.

## Author contributions

XL and YJ designed the study. MW and XL wrote the manuscript. TC, XC, HX and XM edited the paper. YJ supervised the whole paper. All authors contributed to the article and approved the submitted version.

## Funding

This study was supported by Department of Science and Technology of Jilin Provincial (no. 20200201430JC), Jilin Province Development and Reform Commission (no. 2021C043-9), Education Department of Jilin Province (no. JJKH20220194KJ), and the PhD Research Project of Jilin Engineering Normal University (no. BSKJ201923).

## Acknowledgments

We would like to thank the Editage team for English language editing.

## Conflict of interest

The authors declare that the research was conducted in the absence of any commercial or financial relationships that could be construed as a potential conflict of interest.

## Publisher’s note

All claims expressed in this article are solely those of the authors and do not necessarily represent those of their affiliated organizations, or those of the publisher, the editors and the reviewers. Any product that may be evaluated in this article, or claim that may be made by its manufacturer, is not guaranteed or endorsed by the publisher.
